# Correlatıonal effect of sexual myths on sexual qualıty of lıfe in pregnancy: a cross-sectıonal study

**DOI:** 10.1093/sexmed/qfag023

**Published:** 2026-04-20

**Authors:** Gizem Citak, Tugce Gorucu

**Affiliations:** Department of Midwifery, Faculty of Health Sciences, Tokat Gaziosmanpaşa University, Tokat, 60150, Turkey; Graduate School of Education, Tokat Gaziosmanpaşa University, Tokat, 60150, Turkey

**Keywords:** midwifery, pregnancy, sexual myths, sexual quality of life

## Abstract

**Background:**

Sexual myths during pregnancy can shape women’s beliefs about sexuality, increase anxiety, and reduce sexual satisfaction, thereby negatively affecting sexual quality of life.

**Aim:**

This study examined the relationship between sexual myths prevalent during pregnancy and women’s sexual quality of life using a cross-sectional, correlational design.

**Methods:**

Conducted between January and April 2025, this study included 332 pregnant women attending the obstetrics outpatient clinic and wards of a university hospital. Data were collected through face-to-face interviews using a Personal Information Form, the Sexual Myths Scale, and the Sexual Quality of Life Scale.

**Outcomes:**

The primary outcome was sexual quality of life, assessed in relation to the level of endorsement of sexual myths during pregnancy.

**Results:**

Sexual myths varied significantly according to age, education, occupation, spouse’s occupation, marital duration, and pregnancy planning (*P* < .05). Sexual quality of life was also significantly associated with these variables and with pre-pregnancy pain during intercourse (*P* < .05). A strong negative correlation was found between overall sexual myths and sexual quality of life. While the “sexual orientation” subscale showed a positive correlation, the subscales of gender, age and sexuality, sexual behavior, masturbation, and sexual violence were negatively correlated with sexual quality of life (*P* < .005).

**Clinical Implications:**

Midwives should provide evidence-based education and counseling to address sexual myths, thereby supporting healthier sexual experiences and improving well-being during pregnancy.

**Strengths and Limitations:**

This study is among the first to systematically examine this relationship; however, its cross-sectional design limits causal inference, and the findings may not be generalizable beyond the study population.

**Conclusion:**

Integrating sexual health education into routine prenatal care may enhance women’s sexual quality of life during pregnancy.

**Highlight:**

- This study is the first in the Central Black Sea Region of Türkiye to examine the impact of sexual myths on pregnant women’s sexual quality of life.
- Sexual myths were found to be **negatively correlated** with sexual quality of life, indicating that false beliefs increase anxiety and reduce satisfaction during pregnancy.
- Sociodemographic factors such as **age, education, occupation, marital duration, and pregnancy planning** significantly influenced both sexual myths and sexual quality of life.
- The findings emphasize the importance of **evidence-based sexual health education and counseling** by midwives to dispel myths and promote healthier sexual experiences during pregnancy.
- Integrating sexual health education into prenatal care can improve **individual well-being and broader community sexual health outcomes**.

## Introduction

Sexuality is a multifaceted concept encompassing satisfaction and harmony between individuals, shaped by social taboos, rules, and stereotypes, and is considered a basic need for human life.^1–3^ According to the World Health Organization (WHO), sexuality is not merely the absence of disease, dysfunction, or disability; it represents a state of physical, emotional, mental, and social well-being related to sexual expression. In the literature, sexuality is described as a fundamental need that fosters positive relationships between partners, enhances love and self-perception, has mental, physical, and social dimensions, and develops within cultural frameworks.^2^^,^^4^^,^^5^

Sexual myths are widely held but often incorrect beliefs about sexual matters that persist despite lacking scientific evidence.^6–10^ These misconceptions are influenced by cultural, religious, and social factors and can shape attitudes toward sexual health, interpersonal relationships, and sexual behavior.^11–13^ In pregnant women, such beliefs may be associated with changes in sexual desire, arousal, and sexual activity, although these associations do not imply causation.^12^^,^^14^ Research suggests that the prevalence and impact of sexual myths vary across societies and cultural contexts, highlighting the importance of considering sociocultural influences when studying sexual quality of life.^15^^,^^16^ Addressing sexual myths through evidence-based sexual education and counseling may support a more informed understanding of sexuality, which in turn can be associated with better sexual well-being and quality of life, particularly during sensitive life periods such as pregnancy.^17–19^

Pregnancy is a natural period marked by physiological, psychological, and anatomical changes that significantly affect women’s lives.^4^^,^^20^^,^^21^ These changes can influence sexual function and satisfaction.^22^ Although pregnancy provides an opportunity to focus on sexual health, attention to sexual health is often neglected in clinical practice. Studies have shown that sexual activity and the quality of sexual life may vary across trimesters. In the first trimester, changes such as nausea, fatigue, and adaptation to pregnancy may be associated with reduced sexual desire in women, while fathers may experience decreased sexual desire due to fear of harming the baby or miscarriage.^23–25^In the second trimester, as adaptation improves and physical discomforts decrease, sexual desire and activity may increase, though beliefs about fetal development may moderate these behaviors. In the third trimester, factors such as increasing uterine size, fatigue, and fear of impending birth may be associated with reductions in sexual desire and activity.^26–28^

Sexuality in pregnancy has long been subject to misconceptions, myths, and taboos. Social norms may create conflicting expectations regarding sexuality and motherhood, such as the belief that a woman cannot simultaneously be a good mother and a good sexual partner, or that sexual intercourse may harm the fetus.^29^^,^^30^ Cross-cultural studies illustrate these variations: for example, in Turkey, the myth that “the baby feels sexuality” is widely endorsed, while in Iran, beliefs that sexuality during pregnancy is sinful or causes infection are more prevalent.^31^ Prior studies have also shown associations between sexual myths, sexual behavior, and sexual dysfunction in pregnant women.^25^^,^^32^ However, no study has yet examined the association between sexual myths and sexual quality of life during pregnancy in a comprehensive manner.

Despite increasing global attention to sexual health, the literature still lacks sufficient evidence on how culturally rooted sexual myths influence women’s sexual quality of life during pregnancy. Given that pregnancy represents a unique period characterized by significant physiological and psychosocial transformations, exploring this relationship is both timely and relevant to enhance maternal sexual well-being and inform midwifery care practices. In this study, sexual myths refer to widely held but scientifically unfounded beliefs regarding sexual orientation, behavior, satisfaction, intercourse, masturbation, sexual violence, and gender roles, as measured by the Sexual Myths Scale.^33^ Examples include beliefs such as “sexual intercourse during pregnancy harms the baby” or “masturbation negatively affects sexual health.” Therefore, the research question of this study is: Is there a significant relationship between sexual myths and sexual quality of life during pregnancy?

## Material method

### Purpose and type of research

This descriptive, cross-sectional study was conducted to examine the association between sexual myths and sexual quality of life during pregnancy. The study aimed to understand how sexual myths, gender norms, and cultural factors are associated with the sexual quality of life of pregnant women in Turkey.

### Place and time of conducting the research

The study was conducted between January 1, 2025, and April 1, 2025, in the obstetrics outpatient clinics and obstetrics ward of a university hospital. A non-probability convenience sampling method with consecutive recruitment was used. All pregnant women who met the inclusion criteria and attended the obstetrics outpatient clinics and obstetrics ward during the data collection period were consecutively invited to participate on a voluntary basis.

The non-probability sampling method was used in the sample selection of the study and the sample size was calculated using the G*Power 3.1.9.7 program.^34^ Considering Cohen’s (1988) recommendation of a medium effect size, the effect size was determined as f = 0.16, the confidence interval was 80%,^35^ and the margin of error was 5%. As a result of the calculation, it was determined that at least 308 pregnant women were required. Considering a possible 10% data loss, the study was completed with 332 pregnant women.

This study was conducted and reported in accordance with the STROBE (Strengthening the Reporting of Observational Studies in Epidemiology) checklist for cross-sectional studies.

### Participants and eligibility criteria

Pregnant women were eligible to participate in the study if they met the following inclusion criteria: (1) being 18 years of age or older; (2) currently pregnant at any gestational week; (3) married and living with their spouse; (4) able to read and understand Turkish; (5) having no diagnosed psychiatric disorder that could impair comprehension or communication; and (6) voluntarily agreeing to participate in the study.

Women were excluded from the study if they: (1) had a high-risk pregnancy requiring intensive medical management; (2) had a diagnosed severe obstetric complication (eg, placenta previa, preeclampsia with severe features); (3) had a known cognitive impairment; or (4) declined to complete any of the study instruments.

### Data collection tools

*Personal Information Form:* The form, created by the researcher through a literature review, includes a total of 17 questions, including sociodemographic and obstetric characteristics.^32^^,^^36^

*Sexual Quality of Life Scale (SQLS-S):* The Sexual Quality of Life Scale–Short Form was validated by Gölbaşı and Tuğut and consists of 18 items rated on a 6-point Likert scale, with higher scores indicating better sexual quality of life.^37^ Items refer to participants’ sexual experiences during the previous four weeks. Total scores range from 18 to 108 and are converted to a 0–100 scale. Reverse scoring is applied to items 1, 5, 9, 13, and 18. The reported Cronbach’s alpha of the scale is 0.83; in the present study, internal consistency was high (Cronbach’s alpha = 0.85).

*Sexual Myths Scale (SMS):* The Sexual Myths Scale was developed by Gölbaşı et al. and consists of 28 items rated on a 5-point Likert scale ranging from 1 (strongly disagree) to 5 (strongly agree), with higher scores indicating stronger endorsement of sexual myths. The scale includes the following subdimensions: sexual orientation (items 1–5), gender (items 6–11), age and sexuality (items 12–15), sexual behavior (items 16–18), masturbation (items 19–20), sexual violence (items 21–24), sexual intercourse (items 25–26), and sexual satisfaction (items 27–28).^33^ The scale has no cut-off point, and total and subscale scores are calculated by summing item scores. In the original validation study, the overall Cronbach’s alpha was 0.91; in the present study, it was 0.86.

### Data collection procedure

Data were collected through face-to-face interviews conducted by trained midwife researchers. Prior to data collection, the data collectors received standardized training to ensure consistent administration of the questionnaires and to minimize interviewer bias. Interviews were conducted in a private room within the clinic or ward, and no partners or family members were present during data collection to reduce the risk of social desirability bias. Participation was voluntary, and confidentiality was assured.

### Statistical analysis

Statistical analysis of the data was performed using IBM SPSS Statistics for Windows, Version 24.0 (IBM Corp., Armonk, NY, USA). Descriptive statistical measures (mean, standard deviation, minimum and maximum values, and percentages) were used. Normality of continuous variables was assessed using the Shapiro–Wilk test and visual inspection of histograms. The homogeneity of variances was assessed using Levene’s test. Based on these assessments, parametric or non-parametric statistical tests were selected as appropriate. Kruskal–Wallis and Mann–Whitney U tests were applied for non-normally distributed data, and Independent t-test and One-Way ANOVA were applied for normally distributed data. Statistical significance was accepted as *P* < .05. For comparisons involving more than two groups, post-hoc analyses were performed using Tukey’s test for parametric data and Dunn’s test for non-parametric data, with adjustment for multiple comparisons to control the risk of Type I error.

#### Ethical dimension of the research

The study was approved by the Tokat Gaziosmanpaşa University Clinical Research Ethics Committee (Decision No: 01-52, Decision Date: 26.11.2024). In addition, institutional permission was obtained from Tokat Gaziosmanpaşa University Hospital (Decision No: 505779, Decision Date: 05.12.2024). The study was conducted in accordance with the Declaration of Helsinki, and informed consent was obtained from pregnant women who agreed to participate in the study.

## Results

The sociodemographic and obstetric characteristics of the pregnant women are summarized in Table 1.

**Table 1:** Sociodemographic and obstetric characteristics of the participants (N = 332).

Variables		n	%
**Age**	18-22 23-27 28-35 36 and above	16 99 92 125	4.8 29.8 27.7 37.7
**Education Status**	Illiterate Primary school graduate High school graduate Bachelor’s degree	6 44 100 182	1.8 13.3 30.1 54.8
**Occupation**	Housewife Worker Officer Other	176 33 59 64	53.0 9.9 17.8 19.3
**Spouse’s occupation**	Not working Officer Worker Other	5 105 105 117	1.5 31.6 31.6 35.2
**Social security**	No Yes	59 273	17.8 82.2
**Income status**	Less than minimum wage Minimum wage More than minimum wage	30 81 221	9.0 24.4 66.6
**Smoking habit**	No Yes	267 65	80.4 19.6
**Alcohol habit**	No Yes	319 13	96.1 3.9
**Habit-forming substance use**	No Yes	327 5	98.5 1.5
**Presence of chronic disease**	No Yes	290 42	87.3 12.7
**Type of chronic disease (n = 42)**	Thyroid disorders (including goiter) Musculoskeletal system diseases Hepatitis B Asthma/respiratory tract diseases Diabetes Hypertensive disorders Ophthalmologic diseases	15 4 8 4 3 6 2	35.7 9.5 19.3 9.5 7.1 14.2 4.7
**Presence of gynecological disease before pregnancy**	No Yes (What is it?)	259 73	78.0 22.0
**Type of gynecological disorder (n = 73)**	Infection (UTI, Vaginal…) Miscarriage Amenorrhea	48 17 8	65.7 23.8 10.5
**Years of marriage**	1-3 years 4-7 years 8-10 years 11 years and above	120 38 34 140	36.2 11.4 10.2 42.2
**Planned pregnancy**	No Yes	101 231	30.4 69.6
**Gestational week**	1-12 weeks (1st trimester) 13-27 weeks (2nd trimester) 28-40 weeks (3rd trimester)	72 106 154	21.7 31.9 46.4
**Pain during sexual intercourse before pregnancy**	No Yes	243 89	73.2 26.8
**Change in sexual desire during pregnancy**	No change Decreased Increased	137 168 27	41.3 50.6 8.1
**Problems with sexual intercourse during pregnancy**	No Yes	303 29	91.3 8.7

Significant differences were found in the sub-dimensions and total scores of the SMS, as well as the total score of the SQLS-S, based on various sociodemographic factors (*P* < .05). Specifically, age, education level, occupation, spouse’s occupation, smoking status, and presence of chronic disease were significantly associated with the “Sexual Orientation” sub-dimension. Similarly, age, education level, occupation, spouse’s occupation, social security, income level, and presence of chronic disease were related to the “Gender” sub-dimension. The “Age and Sexuality” sub-dimension showed significant associations with age, education level, occupation, spouse’s occupation, and income status. The “Sexual Behavior” sub-dimension was linked to education level, occupation, spouse’s occupation, social security, income status, alcohol, and substance use. Significant relationships were also observed between the “Masturbation” subscale and age, occupation, spouse’s occupation, social security, income status, smoking, substance use, and chronic disease presence. Moreover, the “Sexual Violence” subscale was associated with age, education level, occupation, substance use, and chronic disease. The “Sexual Relationship” subscale correlated with education level, occupation, spouse’s occupation, and chronic disease presence, while the “Sexual Satisfaction” subscale was related to age, occupation, income status, and alcohol use. Additionally, total SMS scores significantly differed by age, educational status, occupation, spouse’s occupation, income level, and substance use, whereas total SQLS-S scores varied with age, educational status, occupation, spouse’s occupation, and income level (Table 2).

**Table 2:** Comparison of total and subscales of Sexual Myths Scale and total Sexual Quality of Life with sociodemographic characteristics.

Variables	Total and subdimensions SQLS									SQLS-S total X-±SD
	**Sexual orientation** **X-±SD**	**Gender** **X-±SD**	**Age and sexuality** **X-±SD**	**Sexual behavior** **X-±SD**	**Masturbation** **X-±SD**	**Sexual violence** **X-±SD**	**Sexual intercourse** **X-±SD**	**Sexual satisfaction** **X-±SD**	**SMS total** **X-±SD**	
**Age** 18-22 23-27 28-35 36 and above	14.87 ± 5.12^abc^ 18.82 ± 4.56^ad^ 20.50 ± 3.57^bde^ 18.72 ± 4.77^ce^	9.18 ± 2.22^ab^ 12.09 ± 3.58^c^ 12.65 ± 4.59^ad^ 14.33 ± 4.91^bcd^	8.12 ± 1.82 8.83 ± 3.69^a^ 9.65 ± 3.02 10.24 ± 3.45^a^	5.81 ± 1.16 6.81 ± 2.82 5.94 ± 2.22 6.18 ± 2.67	4.31 ± 1.19^ab^ 5.81 ± 2.35^a^ 5.42 ± 2.10^c^ 6.29 ± 1.83^bc^	5.87 ± 1.31^abc^ 8.92 ± 3.56^ad^ 8.94 ± 3.30^be^ 10.12 ± 2.81^cde^	6.00 ± 2.19 6.80 ± 2.64 7.10 ± 2.20 7.14 ± 2.03	5.62 ± 1.14^a^ 6.34 ± 2.00 2.83 ± 1.67^a^ 6.31 ± 1.88	59.81 ± 10.33^abc^ 74.47 ± 16.18^a^ 77.07 ± 13.15^b^ 79.37 ± 14.88^c^	89.97 ± 9.55 93.17 ± 12.57^a^ 95.16 ± 9.63^b^ 86.57 ± 12.92^ab^
	***F = 8.347; P < .001***	***F = 9.582; P < .001***	***F = 4.267; P = .006***	KW = 5.920; *P* = .116	***F = 6.247; P < .001***	***F = 10.016; P < .001***	F = 1.482; *P* = .219	***F = 2.772; P < .042***	***F = 9.167; P < .001***	***F = 10.848; P < .001***
**Education Status** Illiterate Primary school graduate High school graduate Bachelor’s degree	19.00 ± 4.38 20.77 ± 3.92^a^ 19.61 ± 3.94 18.34 ± 4.91^a^	17.50 ± 2.73^ab^ 16.68 ± 5.24^cd^ 12.68 ± 4.52^ac^ 12.04 ± 3.83^bd^	12.00 ± 2.19 9.86 ± 2.02^a^ 10.96 ± 3.35^b^ 8.63 ± 3.44^ab^	9.50 ± 2.73^abc^ 6.40 ± 2.12^a^ 6.31 ± 2.61^b^ 6.14 ± 2.57^c^	5.83 ± 0.40 6.13 ± 1.85 6.15 ± 1.84 5.54 ± 2.29	13.00 ± 1.09^abc^ 10.81 ± 2.73^ad^ 9.96 ± 3.42^be^ 8.33 ± 3.02^cde^	8.00 ± 2.19 8.04 ± 1.89^a^ 7.29 ± 1.98^b^ 6.51 ± 2.41^ab^	7.00 ± 1.09 6.63 ± 1.65 6.70 ± 1.88 6.21 ± 1.88	92.00 ± 5.47^ab^ 85.36 ± 9.23^cd^ 79.66 ± 14.75^cae^ 71.80 ± 15.07^bde^	67.00 ± 4.38^abc^ 87.27 ± 9.67^ad^ 91.10 ± 11.83^b^ 92.71 ± 12.44^cd^
	***KW = 11.63; P = .009***	***KW = 34.94; P < .001***	***KW = 38.314; P < .001***	***KW = 8.993; P = .029***	F = 2.195; *P* = .089	***KW = 39.466; P < .001***	***KW = 19.017; P < .001***	KW = 6.275; *P* = .099	***KW = 44.24; P < .001***	***KW = 25.694; P < .001***
**Occupation** Housewife Worker Officer Other	19.57 ± 4.14^a^ 19.51 ± 5.13 19.13 ± 5.08 17.32 ± 4.57^a^	13.89 ± 4.52^ab^ 14.69 ± 5.23^cd^ 11.45 ± 3.99^ac^ 10.82 ± 3.43^bd^	10.48 ± 2.91^ab^ 10.63 ± 4.51^cd^ 7.33 ± 3.20^ac^ 8.51 ± 2.98^bd^	6.68 ± 2.57^a^ 7.12 ± 2.73^b^ 4.91 ± 2.31^ab^ 6.04 ± 2.21	6.12 ± 1.98^a^ 5.78 ± 2.01 5.32 ± 2.12^a^ 5.43 ± 2.34	9.84 ± 3.10^a^ 10.51 ± 3.70^bc^ 7.28 ± 2.74^ab^ 8.70 ± 3.13^c^	7.34 ± 1.94^a^ 7.72 ± 2.28^b^ 5.84 ± 2.93^ab^ 6.64 ± 2.11	6.50 ± 1.68^ab^ 7.93 ± 1.45^acd^ 5.59 ± 2.29^bc^ 6.23 ± 1.54^d^	80.46 ± 12.65^ab^ 83.94 ± 16.31^cd^ 66.90 ± 17.32^ac^ 69.73 ± 12.27^bd^	89.06 ± 12.09^a^ 91.48 ± 11.73 92.77 ± 12.60 94.65 ± 12.37^a^
	***F = 4.042; P = .008***	***F = 12.117; P < .001***	***F = 18.207; P < .001***	***KW = 29.864; P < .001***	***F = 3.103; P = .027***	***F = 12.432; P < .001***	***F = 8.456; P < .001***	***F = 12.913; P < .001***	***F = 22.308; P < .001***	***F = 3.825; P = .010***
**Spouse’s Occupation** Not working Officer Worker Other	24.80 ± 0.44 18.97 ± 4.35 19.73 ± 4.40 18.28 ± 4.76	13.20 ± 1.09 11.38 ± 3.83^ab^ 14.44 ± 4.31^a^ 13.00 ± 4.94^b^	5.60 ± 2.19^a^ 9.15 ± 3.73 10.09 ± 3.35^a^ 9.61 ± 3.05	7.20 ± 1.09 5.65 ± 2.31^a^ 6.64 ± 2.72^a^ 6.49 ± 2.59	2.80 ± 1.09^abc^ 5.55 ± 1.92^ad^ 6.32 ± 2.00^bd^ 5.72 ± 2.23^c^	8.40 ± 2.19 9.00 ± 3.43 9.62 ± 3.14 9.13 ± 3.27	7.80 ± 1.09 6.69 ± 2.52^a^ 7.57 ± 2.22^ab^ 6.66 ± 2.05^b^	6.40 ± 0.54 6.49 ± 2.01 6.60 ± 1.77 6.22 ± 1.80	76.40 ± 0.548^a^ 72.90 ± 15.613^b^ 81.06 ± 14.486^abc^ 75.15 ± 14.81^c^	104.40 ± 4.92^abc^ 91.69 ± 14.15^a^ 89.90 ± 10.35^b^ 90.90 ± 12.25^c^
	***F = 4.881; P = .002***	***KW = 24.618; P < .001***	***KW = 11.296; P = .010***	***KW = 8.144; P = .043***	***KW = 20.234; P < .001***	KW = 3.160; *P* = .368	***KW = 13.250; P = .004***	KW = 3.582; *P* = .310	***KW = 18.613; P < .001***	***KW = 12.079; P = .007***
**Social security** No Yes	19.84 ± 4.54 18.89 ± 4.56	14.44 ± 3.05 12.63 ± 4.73	10.50 ± 3.77 9.35 ± 3.29	7.25 ± 3.49 6.08 ± 2.27	6.62 ± 2.70 5.64 ± 1.91	9.77 ± 3.47 9.12 ± 3.21	7.30 ± 2.50 6.90 ± 2.23	6.35 ± 1.68 6.45 ± 1.88	82.12 ± 13.53 75.08 ± 15.26	89.38 ± 13.54 91.39 ± 12.09
	t = 0.514; *P* = .474	***t = 10.697; P < .001***	t = 2.778; *P* = .097	***t = 12.717; P < .001***	***t = 22.226;*** ***P < .001***	t = 0.005; *P* = .941	t = 1.476; *P* = .225	t = 2.520; *P* = .113	t = 1.809; *P* = .180	t = 1.060; *P* = .304
**Income status** Less than minimum wage Minimum wage More than minimum wage	18.13 ± 4.31 19.55 ± 4.51 19.00 ± 4.62	16.13 ± 2.96^a^ 14.77 ± 5.13^b^ 11.85 ± 4.04^ab^	11.40 ± 2.84^a^ 10.80 ± 2.93^b^ 8.85 ± 3.42^ab^	7.33 ± 2.44^a^ 6.43 ± 2.50 6.09 ± 2.57^a^	6.23 ± 1.69 6.38 ± 2.02^a^ 5.55 ± 2.13^a^	11.33 ± 3.06 10.04 ± 3.22 8.65 ± 3.14	7.50 ± 1.75 7.61 ± 1.82 6.67 ± 2.44	7.20 ± 1.78^a^ 6.24 ± 1.57^a^ 6.39 ± 1.93	85.27 ± 9.45^a^ 81.86 ± 14.94^b^ 73.09 ± 14.88^ab^	87.46 ± 15.43 88.46 ± 11.34^a^ 92.47 ± 12.06^a^
	F = 2.135; *P* = .145	***F = 7.905; P = .005***	***F = 5.630; P = .018***	***F = 10.435; P < .001***	***F = 10.989; P < .001***	F = 1.973; *P* = .161	F = 1.460; *P* = .228	***KW = 9.001; P = .011***	***F = 10.730; P < .001***	***F = 4.580; P = .011***
**Smoking Habit** No Yes	19.26 ± 4.37 18.23 ± 5.25	13.23 ± 4.51 11.80 ± 4.43	9.56 ± 3.42 9.52 ± 3.36	6.23 ± 2.49 6.50 ± 2.84	5.85 ± 2.02 5.64 ± 2.40	9.31 ± 3.12 8.92 ± 3.82	7.16 ± 2.27 6.21 ± 2.18	6.45 ± 1.89 6.35 ± 1.66	77.09 ± 14.80 73.20 ± 16.43	90.40 ± 12.38 93.66 ± 12.01
	***t = 0.370; P < .001***	t = 0.127; *P* = .722	t = 0.122; *P* = .728	t = 2.677; *P* = .103	***t = 8.043; P = .005***	t = 2.288; *P* = .131	t = 0.000; *P* = .999	t = 3.340; *P* = .069	t = 1.061; *P* = .304	t = 0.012; *P* = .912
**Alcohol Habit** No Yes	19.12 ± 4.55 17.53 ± 4.90	12.94 ± 4.46 13.23 ± 6.24	9.49 ± 3.36 11.07 ± 4.29	6.20 ± 2.51 8.30 ± 3.11	5.79 ± 2.11 6.30 ± 1.79	9.24 ± 3.23 9.15 ± 4.14	6.98 ± 2.30 6.76 ± 1.87	6.42 ± 1.87 6.76 ± 1.09	76.21 ± 15.01 79.15 ± 19.49	90.99 ± 12.25 92.30 ± 15.23
	t = 0.267; *P* = .605	t = 3.480; *P* = .063	Z = -0.890; *P* = .374	***Z = -2.019; P = .043***	t = 0.132; *P* = .716	t = 2.003; *P* = .158	t = 0.404; *P* = .525	***t = 4.619; P = .032***	t = 1.324; *P* = .251	t = 1.410; *P* = .236
**Presence of chronic disease** No Yes	19.28 ± 4.41 17.50 ± 5.32	13.01 ± 4.69 12.52 ± 3.20	9.76 ± 3.45 8.14 ± 2.71	6.40 ± 2.56 5.47 ± 2.44	5.85 ± 2.03 5.52 ± 2.54	9.49 ± 3.29 7.45 ± 2.46	7.04 ± 2.19 6.54 ± 2.86	6.51 ± 1.87 5.88 ± 1.59	77.38 ± 15.19 69.05 ± 13.15	90.52 ± 12.34 94.64 ± 12.02
	***t = 4.426; P = .036***	***t = 5.806; P = .017***	t = 3.583; *P* = .059	t = 0.628; *P* = .429	***t = 8.869; P = .003***	***t = 6.568; P = .011***	***t = 10.713; P < .001***	t = 1.866; *P* = .173	t = 2.314; *P* = .129	*t = -0.063; P = .802*

X-: mean. SD: standard deviation, F:One Way ANOVA, KW: Kruskal Wallis, t: Independent Sample T, Z: Man Whitney U, Post-hoc pairwise comparisons were conducted using Tukey’s test (ANOVA) and Dunn’s test (Kruskal–Wallis), with adjustment for multiple comparisons.

When analyzed according to obstetric characteristics, significant differences were also identified in the sub-dimensions and total scores of the SMS and total SQLS-Scores (*P* < .05). The “Sexual Orientation” sub-dimension was associated with pregnancy planning, pre-pregnancy pain during intercourse, changes in sexual desire during pregnancy, and sexual relationship problems. The “Gender” sub-dimension correlated with years of marriage, pre-pregnancy pain, and relationship problems during pregnancy. “Age and Sexuality” was significantly related to years of marriage and changes in sexual desire during pregnancy. The “Sexual Behavior” sub-dimension was associated with gestational age, changes in desire, and relationship issues. The “Masturbation” sub-dimension showed significant associations with gynecological disease history, years of marriage, and gestational age. The “Sexual Violence” sub-dimension correlated with years of marriage and changes in sexual desire. The “Sexual Relationship” sub-dimension was related to gynecological disease, years of marriage, pregnancy planning, gestational age, and history of painful intercourse. Lastly, the “Sexual Satisfaction” sub-dimension differed significantly with years of marriage, pregnancy planning, and relationship problems. Total SMS scores were significantly associated with years of marriage and pregnancy planning, while total SQLS-S scores correlated with years of marriage and pre-pregnancy painful intercourse (Table 3).

**Table 3:** Comparison of total and subscales of Sexual Myths Scale and total Sexual Quality of Life with obstetric characteristics.

Variables	Total and subdimensions of SMS									SQLS-S total X-±SD
	Sexual orientation X-±SD	Gender X-±SD	Age and sexuality X-±SD	Sexual behavior X-±SD	Masturbation X-±SD	Sexual violence X-±SD	Sexual intercourse X-±SD	Sexual satisfaction X-±SD	SMS total X-±SD	
**Presence of gynecological disease before pregnancy** No Yes	19.17 ± 4.46 18.65 ± 4.94	12.89 ± 4.75 13.13 ± 3.67	9.69 ± 3.46 9.06 ± 3.18	6.29 ± 2.58 6.27 ± 2.51	5.79 ± 2.15 5.89 ± 1.92	9.24 ± 3.29 9.20 ± 3.21	7.03 ± 2.16 6.79 ± 2.68	6.53 ± 1.84 6.08 ± 1.84	76.67 ± 15.47 75.10 ± 14.16	91.94 ± 12.43 87.82 ± 11.62
	Z = -0.684; *P* = .494	Z = -1.143; *P* = .253	t = 0.365; *P* = .546	t = 0.365; *P* = .546	***t = -5.693; P = .018***	t = 2.414; *P* = .121	***t = 12.072; P < .001***	t = 0.039; *P* = .843	Z = -0.724; *P* = .469	t = 3.264; *P* = .072
**Years of marriage** 1-3 Years 4-7 Years 8-10 Years 11 Years and above	18.53 ± 4.82 19.02 ± 4.42 19.44 ± 4.74 19.42 ± 4.34	11.80 ± 3.81^a^ 11.52 ± 3.67^b^ 13.97 ± 3.46 14.07 ± 5.17^ab^	8.74 ± 3.44^a^ 8.71 ± 3.30^b^ 10.23 ± 3.03 10.32 ± 3.30^ab^	6.32 ± 2.63 5.78 ± 2.62 6.97 ± 3.41 6.22 ± 2.22	5.53 ± 2.39^a^ 5.34 ± 1.97^b^ 6.67 ± 2.11^ab^ 5.97 ± 1.78	8.45 ± 3.55^ab^ 7.76 ± 3.14^cd^ 10.23 ± 3.08^ac^ 10.07 ± 2.77^bd^	6.80 ± 2.74^a^ 6.47 ± 1.94^b^ 8.29 ± 1.52^abc^ 6.95 ± 1.98^c^	6.22 ± 2.03^a^ 6.21 ± 1.06^b^ 8.00 ± 1.39^abc^ 6.29 ± 1.78^c^	72.40 ± 16.97^ab^ 70.84 ± 12.74^cd^ 83.82 ± 7.76^ac^ 79.35 ± 14.17^bd^	92.57 ± 11.84^a^ 97.31 ± 8.01^bc^ 88.73 ± 15.58^b^ 88.58 ± 12.20^ac^
	F = 0.913; *P* = .435	***F = 7.739; P < .001***	***F = 6.187; P < .001***	F = 1.317; *P* = .269	***F = 3.627; P = .013***	***F = 9.685; P < .001***	***F = 4.772; P = .003***	***F = 9.788; P < .001***	***F = 9.627; P < .001***	***F = 6.412; P < .001***
**Planned pregnancy** No Yes	18.36 ± 4.48 19.36 ± 4.58	14.51 ± 5.05 12.26 ± 4.11	10.08 ± 2.99 9.32 ± 3.55	6.44 ± 2.89 6.22 ± 2.41	6.53 ± 1.98 5.50 ± 2.07	10.35 ± 3.38 8.74 ± 3.10	7.33 ± 1.99 6.82 ± 2.39	6.52 ± 1.72 6.39 ± 1.90	80.16 ± 15.93 74.64 ± 14.57	87.14 ± 13.15 92.74 ± 11.62
	***t = 4.385; P = .038***	t = 0.987; *P* = .322	t = 0.092; *P* = .762	t = 0.076; *P* = .783	t = 2.640; *P* = .106	t = 1.855; *P* = .175	***t = 11.307; P < .001***	***t = 12.000; P < .001***	***t = 6.539; P = .012***	t = 3.347; *P* = .068
**Gestational week** 1-12 weeks (1st trimester) 13-27 weeks (2nd trimester) 28-40 weeks (3rd trimester)	19.06 ± 4.84 18.57 ± 4.60 19.38 ± 4.41	13.34 ± 4.63 12.61 ± 4.33 13.00 ± 4.62	9.62 ± 3.00 9.68 ± 3.54 9.44 ± 3.50	7.13 ± 2.73^a^ 6.43 ± 2.85 5.79 ± 2.13^a^	6.20 ± 1.86^a^ 5.11 ± 2.18^ab^ 6.11 ± 2.04^b^	9.68 ± 3.53 8.65 ± 3.31 9.43 ± 3.07	7.00 ± 2.23 6.51 ± 2.48^a^ 7.28 ± 2.12^a^	6.13 ± 2.07 6.59 ± 1.77 6.46 ± 1.79	78.20 ± 15.94 74.18 ± 15.61 76.92 ± 14.44	89.81 ± 12.77 91.06 ± 13.67 91.59 ± 11.21
	F = 0.996; *P* = .371	F = 0.577; *P* = .562	F = 0.181; *P* = .835	***F = 7.267; P < .001***	***F = 9.181; P < .001***	F = 2.672; *P* = .071	***F = 3.585; P = .029***	F = 1.330; *P* = .266	F = 1.728; *P* = .179	F = 0.506; *P* = .603
**Pain during sexual desire during pregnancy** No Yes	19.21 ± 4.31 18.64 ± 5.22	13.56 ± 4.50 11.28 ± 4.20	9.63 ± 3.38 9.35 ± 3.49	6.30 ± 2.68 6.25 ± 2.22	6.12 ± 2.13 4.97 ± 1.77	9.47 ± 3.29 8.59 ± 3.12	6.90 ± 2.21 7.17 ± 2.48	6.44 ± 1.81 6.40 ± 1.96	77.65 ± 15.24 72.69 ± 14.50	90.75 ± 12.80 91.82 ± 11.08
	***t = 8.062; P = .005***	***Z = -4.401; P < .001***	t = 1.566; *P* = .212	t = 2.150; *P* = .144	t = 2.233; *P* = .136	t = 0.971; *P* = .325	***t = 4.470; P = .035***	t = 1.778; *P* = .183	t = 0.078; *P* = .827	***t = 4.349; P = .038***
**Change in sexual desire during pregnancy** No change Decreased Increased	18.22 ± 4.29^a^ 19.76 ± 4.57^a^ 18.88 ± 5.27	13.36 ± 4.74 12.73 ± 4.64 12.22 ± 3.71	8.93 ± 3.08^a^ 10.07 ± 3.50^a^ 9.55 ± 3.93	6.32 ± 2.72^a^ 6.00 ± 2.35^b^ 7.88 ± 2.47^ab^	5.73 ± 2.09 5.92 ± 2.01 5.55 ± 2.67	9.15 ± 3.50^a^ 9.61 ± 3.09^b^ 7.33 ± 2.35^ab^	6.72 ± 2.10 7.26 ± 2.36 6.44 ± 2.50	6.29 ± 1.79 6.51 ± 1.93 6.66 ± 1.59	74.76 ± 13.94 77.88 ± 15.69 74.55 ± 17.50	91.73 ± 12.01 89.91 ± 12.99 94.55 ± 9.05
	***F = 4.399; P = .013***	F = 1.117; *P* = .328	***F = 4.282; P = .015***	***F = 6.539; P = .002***	F = 0.518; *P* = .596	***F = 5.896; P = .003***	F = 2.921; *P* = .055	F = 0.763; *P* = .467	F = 1.801; *P* = .167	F = 2.022; *P* = .134
**Problems with sexual intercourse during pregnancy** No Yes	19.12 ± 4.50 18.34 ± 5.21	13.05 ± 4.66 11.82 ± 2.49	9.68 ± 3.38 8.24 ± 3.48	6.37 ± 2.64 5.37 ± 1.29	5.83 ± 2.07 5.58 ± 2.44	9.22 ± 3.30 9.34 ± 2.97	6.90 ± 2.21 7.79 ± 2.87	6.33 ± 1.81 7.48 ± 1.95	76.55 ± 15.39 74.00 ± 12.86	90.81 ± 12.64 93.37 ± 8.80
	t = 3.548; *P* = .061	***t = 9.983; P = .002***	t = 0.177; *P* = .674	***t = 8.985; P = .003***	t = 2.423; *P* = .121	t = 1.932; *P* = .165	t = 1.805; *P* = .180	***Z = -3.592; P = .005***	t = 2.539; *P* = .112	t = 3.591; *P* = .059

X-: mean. SD: standard deviation, F:One Way ANOVA, KW: Kruskal Wallis H test, t: Independent Sample T, Z: Mann–Whitney U, Post-hoc pairwise comparisons were conducted using Tukey’s test (ANOVA) and Dunn’s test (Kruskal–Wallis), with adjustment for multiple comparisons.

As shown in Table 4, a significant negative correlation was observed between the total scores of the SMS and SQLS (r = –0.274, *P* = .001). Additionally, sub-dimensions such as Gender, Age and Sexuality, Sexual Behavior, Masturbation, and Sexual Violence showed significant negative correlations with sexual quality of life scores (*P* < .05). Conversely, the Sexual Orientation sub-dimension was positively correlated with sexual quality of life.

**Table 4:** The relationship between the total and sub scale scores of the Sexual Myths Scale and the total scores of Sexual Quality of Life.

		1	2	3	4	5	6	7	8	9	10
**Sexual Quality of Life Scale Total(1)**	**rp**	. 1	***−0.274*** ***0.001***	***0.119*** ***0.031***	***−0.362*** ***0.001***	***−0.239*** ***0.001***	***−0.263*** ***0.001***	***−0.192*** ***0.001***	***−0.372*** ***0.001***	*−0.036* *0.508*	*0.069* *0.210*
**Sexual Myth Scale Total (2)**	**rp**		*1*	***0.547*** ***0.001***	***0.705*** ***0.001***	***0.663*** ***0.001***	***0.615*** ***0.001***	***0.522*** ***0.001***	***0.721*** ***0.001***	***0.564*** ***0.001***	***0.491*** ***0.001***
**Sexual Orientation Subscale (3)**	**rp**			*1*	***0.186*** ***0.001***	***0.110*** ***0.045***	*0.097* *0.079*	***0.190*** ***0.001***	***0.130*** ***0.017***	***0.404*** ***0.001***	***0.283*** ***0.001***
**Gender Subdimension(4)**	**rp**				1	***0.367*** ***0.001***	***0.478*** ***0.001***	***0.304*** ***0.001***	***0.502*** ***0.001***	***0.121*** ***0.027***	***0.157*** ***0.004***
**Age and Sexuality Subdimension(5)**	**rp**					1	***0.442*** ***0.001***	***0.354*** ***0.001***	***0.508*** ***0.001***	***0.221*** ***0.001***	***0.246*** ***0.001***
**Sexual Behavior Subdimension(6)**	**rp**						1	***0.220*** ***0.001***	***0.397*** ***0.001***	***0.213*** ***0.001***	***0.223*** ***0.001***
**Masturbation Subscale (7)**	**rp**							1	***0.372*** ***0.001***	***0.213*** ***0.001***	0.059 0.281
**Sexual Violence Subdimension(8)**	**r** **p**								1	***0.349*** ***0.001***	***0.258*** ***0.001***
**Sexual** **Relationship Subscale(9)**	**r** **p**									1	***0.534*** ***0.001***
**Sexual Satisfaction Subscale (10)**	**r** **p**										1

## Discussion

In our study, statistically significant differences were found between the total scores of the SQLS-S and variables such as age, educational status, occupation, spouse’s occupation, income status, duration of marriage, and experiencing pain during sexual intercourse before pregnancy (*P* < .005). These sociodemographic and relational factors may influence sexual quality of life by shaping women’s access to information, communication patterns within the relationship, and perceptions of sexuality during pregnancy.

Studies conducted in Iran, a society characterized by strong religious norms and traditional gender roles, indicate that sexuality is often regulated through cultural taboos, moral expectations, and gender-specific behavioral norms. Sexuality-related topics are frequently considered private or restricted, and premarital sexual behaviors are socially and legally discouraged.^38^ In this context, limited sexual education and the moralization of sexuality may contribute to misconceptions, anxiety, and avoidance behaviors, particularly during sensitive periods such as pregnancy. These findings suggest that in culturally conservative settings, sociocultural and religious norms can shape sexual beliefs and practices, thereby influencing sexual quality of life. Similar mechanisms may also be relevant in other societies with strong traditional and religious values, including Turkey, highlighting the importance of culturally sensitive interpretations when examining sexual myths and sexual well-being during pregnancy. Within this framework, the observed associations between sociodemographic characteristics and sexual quality of life in our study may reflect the internalization of culturally shaped sexual norms rather than individual characteristics alone.

These findings align with those of a 2023 study reporting that pregnant women with a marriage duration of less than five years, high economic status, and education level of high school or above had significantly higher sexual quality of life and happiness levels.^39^ Higher education may contribute to improved sexual quality of life by facilitating greater sexual health literacy, reducing misconceptions, and enabling more open communication with partners. Similarly, Acısu (2019)^40^ found significant relationships between sexual quality of life and age at marriage, age at first pregnancy, and gestational week. Kodaz (2013)^41^ also reported that sexual intercourse frequency increased alongside sexual quality of life scores. Taken together, these findings suggest that sexual quality of life is not only a reflection of sexual activity but also a multidimensional construct influenced by relational dynamics, physical comfort, and psychosocial well-being.

However, some studies report conflicting findings. For example, Şolt Kırca and Dağlı (2023)^21^ found no significant differences between sexual quality of life and factors such as pregnancy week, education level, spouse’s education level, family type, income level, employment status, or pregnancy planning status, suggesting that sexual quality of life may not be uniformly shaped by sociodemographic variables across different samples. Yet, they reported a significant association between the number of pregnancies and sexual quality of life.

Similarly, Hacıköylü and Öztürk (2023)^42^ observed no significant differences in sexual quality of life across sociodemographic groups but noted generally high sexual quality of life scores. Conversely, Şahingöz (2021)^43^ reported significant associations between sexual quality of life and variables such as pregnancy desire, nulliparity, baby’s sex, informing the spouse, and regular pregnancy follow-up, with scores decreasing as gestational week progressed. Bilge et al. (2021)^28^ noted that sexuality is particularly affected in the first and last trimesters of pregnancy, with pain during intercourse and sexual communication problems commonly reported. Taken together, these divergent findings indicate that sexual quality of life during pregnancy is a multidimensional and context-dependent construct, shaped by the interaction of biological changes, relational dynamics, and sociocultural norms. Differences in sample characteristics, cultural context, and timing of data collection may partly explain inconsistencies across studies. In this context, our findings support the need for individualized and culturally sensitive counseling on sexual quality of life during prenatal care, rather than a uniform approach, to promote both maternal well-being and healthy couple relationships.

Furthermore, our study demonstrated that both total scores and sub-dimensions of the CPS varied significantly according to a wide range of sociodemographic and obstetric variables, including age, education level, occupation, duration of marriage, spouse’s occupation, social security status, income level, presence of chronic disease, pre-pregnancy illness, gestational week, pregnancy planning, pain during sexual intercourse before pregnancy, changes in sexual desire during pregnancy, and the presence of sexual problems. Rather than representing isolated associations, these findings suggest that sexual myths during pregnancy are embedded within broader socioeconomic, cultural, and relational contexts that shape women’s beliefs about sexuality.

In particular, myths centered on fetal harm, premature birth, or religious prohibitions appear to function as cognitive barriers that promote fear-based avoidance of sexual activity, thereby increasing sexual problems and potentially diminishing sexual quality of life. Özçoban and Dilcen (2022)^44^ reported that many pregnant women experience sexual problems during the first trimester due to beliefs that sexual intercourse may harm the baby, supporting the notion that myth-driven anxiety plays a central role in shaping sexual behavior during pregnancy. Interestingly, Öztürk Altınayak and Özkan (2023)^45^ found that sexual myths increased with higher educational levels of both the pregnant woman and her partner, indicating that formal education alone may be insufficient to counteract culturally embedded misconceptions without targeted sexual health education.

Similarly, Güney and Bal (2023)^46^ and Ünal (2019)^47^ identified age, education, income level, and duration of marriage as key determinants of sexual myths. Taken together, these findings highlight that sexual myths are not merely knowledge deficits but are reinforced by cultural norms, gender roles, and moral frameworks surrounding pregnancy and sexuality. Moreover, Ninivaggio et al. (2016)^48^ demonstrated that such false beliefs may impair sexual function, suggesting a pathway through which myths translate into tangible sexual health outcomes. Consistent with this evidence, our findings confirm that sexual myths during pregnancy are shaped by multiple sociodemographic and obstetric factors and may exert their influence through cognitive and behavioral mechanisms. Therefore, identifying and addressing these myths through evidence-based counseling and prenatal sexual health education should be considered a core component of midwifery care aimed at promoting healthy sexual lives among pregnant women and their partners.

A strong and statistically significant negative correlation was identified between the total scores of the SMS and the SQLS-S among pregnant women (*P* < .005), indicating that greater endorsement of sexual myths is associated with poorer sexual quality of life during pregnancy. Rather than reflecting a simple inverse association, this finding suggests a cognitive–behavioral pathway in which myth-based beliefs increase anxiety, promote avoidance of intimacy, and ultimately undermine sexual satisfaction.

Previous studies provide support for this interpretation. Şahingöz (2021)^43^ reported higher sexual quality of life among individuals who did not endorse sexual myths, while Çelimli Oruç (2022)^36^ found that sexual quality of life declined as negative attitudes and beliefs about sexuality during pregnancy increased. Aşçı and Gökdemir (2021)^49^ similarly demonstrated that stronger belief in sexual myths was associated with more negative sexual attitudes and beliefs. Collectively, these findings reinforce the notion that sexual myths function as maladaptive cognitive schemas that distort perceptions of sexual safety and pleasure during pregnancy. Studies focusing on sexual satisfaction have likewise shown that increasing endorsement of sexual myths is linked to reduced sexual satisfaction.^49^

At the sub-dimension level, our results further clarify this relationship. While the sexual orientation sub-dimension was positively associated with sexual quality of life, the gender, age and sexuality, sexual behavior, masturbation, and sexual violence sub-dimensions demonstrated significant negative associations (*P* < .005). This pattern suggests that myths grounded in gender norms, bodily control, and moralized views of sexuality are particularly detrimental to intimacy and sexual well-being during pregnancy. In contrast, greater awareness and acceptance of individual and identity-related aspects of sexuality, such as sexual orientation, may foster openness, communication, and emotional closeness, thereby enhancing sexual quality of life.

Taken together, these findings indicate that false beliefs about sexuality during pregnancy can directly impair sexual satisfaction and intimacy, whereas accurate knowledge and self-awareness may serve protective roles. Accordingly, tailored, evidence-based education and counseling interventions aimed at challenging sexual myths should be integrated into prenatal care, as socially and culturally constructed beliefs about sexuality play a central role in shaping sexual health outcomes during pregnancy.

## Strengths and limitations

This study has several strengths, including a relatively large sample, the use of validated instruments (the SMS and the SQLS–S Form), and face-to-face data collection, which ensured accurate understanding of the items. It systematically examined the relationship between sexual myths and sexual quality of life, providing valuable insights into the Turkish sociocultural context. Several limitations should be noted: the sample was recruited from a single university hospital, which may limit generalizability, and sexual myths and attitudes are influenced by cultural, religious, and social norms, which may differ across societies. Despite these limitations, the underlying mechanisms identified such as myth-based beliefs increasing anxiety, promoting sexual avoidance, and impairing intimacy are consistent with broader theoretical frameworks and may offer insights applicable beyond this cultural context, although cross-cultural studies are needed to confirm transferability.

## Conclusion and recommendations

This study highlights that sexual myths during pregnancy particularly those related to gender, sexual behavior, age, and sexuality negatively affect women’s sexual quality of life. Increased belief in these myths is linked to decreased sexual satisfaction and overall well-being, reflecting the profound influence of cultural norms on both intimate health and public health.

From a midwifery and public health perspective, addressing these myths through accurate, respectful, and culturally sensitive education should be a fundamental part of prenatal care. Integrating comprehensive sexual health counseling into routine midwifery services not only supports individual women’s well-being and relationship satisfaction but also contributes to broader public health goals by promoting healthy sexual attitudes and reducing misconceptions at the community level.

## Supplementary Material

Supplementary-Material_qfag023

## Data Availability

https://doi.org/10.5281/zenodo.15465465
