# Supplementary material for: Correlatıonal effect of sexual myths on sexual qualıty of lıfe in pregnancy: a cross-sectıonal study
Source: Sex Med. 2026 Apr 20;14(3):qfag023. doi: 10.1093/sexmed/qfag023 (PMC13092729; doi:10.1093/sexmed/qfag023)
Supplement: Supplementary-Material_qfag023 [file supplementary-material_qfag023.zip › Intihal_Proje_Detaylari_qfag023.pdf]

intihal.net

Yazar : **Sayfa Sayısı :** 17 **Kelime Sayısı :** 5919 **Karakter Sayısı :** 35439

Benzerlik raporu ayarları : **Asgari Kelime Sayısı :** 9 **Atıf ve Kaynakça:** Atıf ve Kaynakça Çıkarıldı.

ORİJİNALLİK RAPORU

BENZERLİK ENDEKSİ	İNTERNET KAYNAKLARI	YAYINLAR	ÖĞRENCİ ÖDEVLERİ
%7	-	-	-

BİRİNCİL KAYNAKLAR

1	null -<br>**Proceedings from North American Symposium on Knowledge Organization, Vol. 3**	<%1
2	Middle Black Sea Journal of Health Science (Dergipark) -<br>**Mode of Delivery and Number of Children Effect on Sexual Function**	<%1
3	Georg Thieme Verlag KG -<br>**Pituitary Disease in Pregnancy: Special Aspects of Diagnosis and Treatment?**	<%1
4	Cyprus Journal of Medical Sciences -<br>**Turkish Expectant Fathers' Experiences of Sexual Life During Pregnancy: A Qualitative Study**	<%1
5	Uniwersytet Lodzki (University of Lodz) -<br>**#Underboobselfie, #aftersexselfie, #bikinibridgeselfie... La sexualité reste-t-elle encore un tabou dans les réseaux sociaux ?**	<%1
6	Ege Üniversitesi -<br>**Kemoterapi tedavisi alan çocuklarda oral mukozitin yaşam kalitesine etkisi**	<%1
7	Necmettin Erbakan Üniversitesi -<br>**Gebelikte cinsel mitlerin cinsel yaşam kalitesi üzerine etkisi**	<%1
8	Gümüşhane Üniversitesi Sağlık Bilimleri Dergisi -<br>**Health Professionals' Attitudes Towards Lesbian and Gay Individuals, and Levels of Homophobia and Empathy: A Case of Turkey**	<%1
9	Türkiye Diyabet ve Obezite Dergisi -<br>**Tip 2 Diyabetli Bireylerin e-Sağlık Okuryazarlık Düzeyi ile Dijital Ortamdan Sağlık Bilgisi Edinme ve Teyit Davranışları Arasındaki İlişki: Bir Kamu Hastanesi Örneği**	<%1
10	6.INTERNATIONAL EURASIAN EDUCATIONAL RESEARCH CONGRESS -<br>**LİSE ÖĞRENCİLERİNİN ÇEVRESEL TUTUMLARI (ÇANKIRI İLİ ÖRNEĞİ)**	<%1
11	Archives of health science and research (Online) -<br>**The Situation of Intensive Care Nurses Using Evidence-Based Guidelines in Preventing Central Venous Catheter-Related Infections**	<%1
12	Türk Eğitim Bilimleri Dergisi -<br>**Türkiye'de Yapılan Lisansüstü Tezlerin Okul Öncesi Dönem Çocuklarının Gelişimlerine Etkisi: Bir Meta Analiz Çalışması**	<%1
13	Koç Üniversitesi -<br>**Yenidoğan yoğun bakım hemşirelerinin bireyselleştirilmiş gelişimsel bakım davranışlarının hemşirelerin yaşam kaliteleri üzerine etkisi**	<%1

14	Galenos Publishing -<br>**Evaluation of the efficacy of injectable platelet-rich fibrin in genitourinary syndrome of menopause**	<%1
15	Informa UK Limited -<br>**Does Infertility Affect the Sexual Function and Sexual Quality of Life of Women? A Case Control Study**	<%1
16	Springer Science and Business Media LLC -<br>**Sexual Myths and Sexual Health Knowledge Levels of Turkish University Students**	<%1
17	Seçil Yıldız, Nefise Semra Erkan -<br>**A Scale Development Study to Determine Preschool Teachers' Perception of Gender Roles in the Classroom**	<%1
18	Başkent Üniversitesi -<br>**The moderating effect of social emotional learning skills in the relationship between schema child modes and violence tendency**	<%1
19	MediHealth Academy Yayıncılık -<br>**Evaluation of pressure ulcer development and risk factors in COVID-19 patients followed in the ICU**	<%1
20	Springer-Verlag -<br>**Power Doppler ultrasonography is useful for assessing disease activity and predicting joint destruction in rheumatoid arthritis patients receiving tocilizumab—preliminary data**	<%1
21	Gilmer, Debbie, McElroy, Meg -<br>**Student Outcomes: Studying the Effectiveness of Transition in Maine. Final Report.**	<%1
22	Asian Online Journal Publishing Group. 244 Fifth Avenue Suite D42, New York, NY 10001. Fax: 212-591-6094; e-mail: info@asianonlinejournals.com; Web site: http://www.asianonlinejournals.com -<br>**The Relationship of Mental Toughness and Emotional Eating: The Example of a Female Wrestler**	<%1
23	-	<%1
24	SAGE Publications -<br>**Predictors of Clinical Performance Among Emergency Nurses: A Cross-Sectional Study**	<%1
25	Unknown -<br>**LEVERAGING ENTREPRENEURSHIP EDUCATION IN UPSCALING INFORMAL ECONOMY OF NIGERIA**	<%1
26	0 -<br>**Obstructive Sleep Apnea in Children with Syndromic Craniosynostosis**	<%1
27	İnönü üniversitesi Sağlık Hizmetleri Meslek Yüksek Okulu Dergisi -<br>**İLK VE ACİL YARDIM PROGRAMI ÖĞRENCİLERİNİN MESLEKİ UYGULAMALARI YAPMA VE YETERLİ HİSSETME DURUMLARININ BELİRLENMESİ**	<%1
28	Selçuk Üniversitesi -<br>**Sağlık sistemlerine güvensizliğin hekim sahiplenmeye etkisi**	<%1
29	Hasan ŞAHAN -<br>**Effect of Athletes Social Intelligence Levels on Decision Making**	<%1
30	MDPI AG -<br>**Cleft Candidate Genes and Their Products in Human Unilateral Cleft Lip Tissue**	<%1
31	International Journal of Sport Culture and Science -<br>**Netlessfobi ile Serbest Zaman Doyumu Arasındaki İlişki: Spor Bilimleri Fakültesi Öğrencileri Üzerine Bir Araştırma**	<%1

32	Ege Üniversitesi -<br>**Yaşlı bireylerin uyguladıkları manevi uygulamalar ve manevi iyi oluşun sağlık algısı üzerine etkisi**	<%1
33	Wiley -<br>**The potential forFusarium oxysporumf. sp.fragariae, cause of fusarium wilt of strawberry, to colonize organic matter in soil and persist through anaerobic soil disinfestation**	<%1
34	Eurasian Journal of Health Sciences -<br>**The Effect of Information and Coping with Anxiety Training Given to Women before Hysterectomy on their Level of Anxiety: A Post-Test Randomized Controlled Experimental Study**	<%1
35	Elsevier BV -<br>**The effect of diluted lavender oil inhalation on pain development during vascular access among patients undergoing haemodialysis**	<%1
