# Supplementary material for: Correlatıonal effect of sexual myths on sexual qualıty of lıfe in pregnancy: a cross-sectıonal study
Source: Sex Med. 2026 Apr 20;14(3):qfag023. doi: 10.1093/sexmed/qfag023 (PMC13092729; doi:10.1093/sexmed/qfag023)
Supplement: Supplementary-Material_qfag023 [file supplementary-material_qfag023.zip › KURUM_qfag023.pdf]

T.C.
TOKAT GAZİOSMANPAŞA ÜNİVERSİTESİ
Sağlık Araştırma ve Uygulama Merkezi Müdürlüğü

Sayı : E-21979232-044-505779 05.12.2024
Konu : Anket Çalışma İzni

SAĞLIK BİLİMLERİ FAKÜLTESİ DEKANLIĞINA

İlgi : 04.12.2024 Tarihli, 504955 sayılı yazı.

İlgi yazınızda ekte tabloda belirtildiği ifade edilen çalışmalardan 2 tanesi için dosya hazırlanmış olduğu görülmüştür. Bu nedenle Dr. Öğr. Üyesi Gizem ÇITAK'ın,"Gebelikte Cinsel Mitlerin Cinsel Yaşam Kalitesine Etkileri" ve "Gebelikte Cinsel Mitlerin Cinsel Yaşam Kalitesine Etkileri" başlıklı çalışmaları için 01.01.2025- 01.04.2025 tarihleri arasında Hastanemiz kadın doğum poliklinikleri ve kadın doğum servislerinde gebelerle yüz-yüze görüşülerek yürütülmesi uygun görülmüştür. Bundan sonra yapılacak çalışmalar için yazınızda hangi başlıklı çalışmalarınız için izin istendiğinin yazılarak bildirilmesi hususunda;

Gereğini bilgilerinize arz ederim.

Doç. Dr. Muzaffer KATAR
Merkez Müdür Yardımcısı

Bu belge, güvenli elektronik imza ile imzalanmıştır.

Belge Doğrulama Kodu :BSPZS4ND18 Pin Kodu :66352 Belge Takip Adresi : https://turkiye.gov.tr/ebd?eK=5695&eD=BSPZS4ND18&eS=505779

Adres:Tokat Gaziosmanpaşa Üniversitesi Ali Şevki Erek Yerleşkesi Merkez Tokat
Telefon:2129500 Faks:2122142
e-Posta:hastane@gop.edu.tr Web:http://hastane.gop.edu.tr
Kep Adresi:gaziosmanpasa.universitesi@hs03.kep.tr

Bilgi için: Elif Mazman
Unvanı: Sağlık Teknikeri

Bu belge,güvenli elektronik imza ile imzalanmıştır.
