# Supplementary material for: Correlatıonal effect of sexual myths on sexual qualıty of lıfe in pregnancy: a cross-sectıonal study
Source: Sex Med. 2026 Apr 20;14(3):qfag023. doi: 10.1093/sexmed/qfag023 (PMC13092729; doi:10.1093/sexmed/qfag023)
Supplement: Supplementary-Material_qfag023 [file supplementary-material_qfag023.zip › STROBE.pdf]

STROBE Statement—checklist of items that should be included in reports of observational studies

| | Item No | Recommendation |
|---|---|---|
| **Title and abstract** | 1 | Title clearly states "A Cross-Sectional Study"; abstract summarizes objectives, methods, results, and conclusions (see Abstract, lines 1–25). |
| **Introduction** | | |
| Background/rationale | 2 | Explain the scientific background and rationale for the investigation being reported State specific objectives, including any prespecified hypotheses |
| Objectives | 3 | Clearly stated in the last paragraph of the Introduction: "This study was planned to determine the effect of sexual myths on the quality of sexual life during pregnancy." |
| **Methods** | | |
| Study design | 4 | Described in "Purpose and Type of Research" section: descriptive, cross-sectional, correlational design (Methods, lines 1–5). |
| Setting | 5 | Provided in "Place and Time of Conducting the Research" section (Methods, lines 6–16). |
| Participants | 6 | *Described in "Place and Time of Conducting the Research" section; non-probability sampling, inclusion of 332 pregnant women (Methods, lines 6–16).* |
| Variables | 7 | Sexual myths (SMS) and sexual quality of life (SQLS-S) defined, including subscales and scoring (Methods, Data Collection Tools, lines 17–65). |
| Data sources/ measurement | 8* | Described in "Data Collection Tools" with validated scales, scoring, and reliability coefficients (Methods, lines 17–65). |
| Bias | 9 | Sample selection, standardised questionnaires, face-to-face interviews, trained data collectors, and Cronbach's alpha values reported to ensure reliability (Methods, lines 6–65). |
| Study size | 10 | Sample size calculated using G*Power software, effect size, confidence interval, and expected data loss provided (Methods, lines 6–16). |
| Quantitative variables | 11 | Total and subscale scores of SMS and SQLS-S described; scoring system and calculation provided (Methods, Data Collection Tools, lines 17–65). |
| Statistical methods | 12 | Statistical tests (Kruskal-Wallis, Mann-Whitney U, t-test, ANOVA, Spearman correlation) reported, software specified (SPSS 24.0, IBM Corp.) (Methods, Statistical Analysis, lines 66–75). |

| **Results** | | |
|---|---|---|
| Participants | 13* | 332 participants included; sample size calculation and accounting for data loss described (Methods, lines 6–16). |
| Descriptive data | 14* | Sociodemographic and obstetric characteristics summarized in Results, Table 1 (Results, lines 76–100). |
| Outcome data | 15* | *Sexual myths and sexual quality of life scores reported with mean ± SD; subscale scores and total scores presented (Results, lines 101–145).* |
| Main results | 16 | Subscale analyses of SMS with correlations to SQLS-S presented (Results, lines 130–145). |
| Other analyses | 17 | Associations between sociodemographic/obstetric factors and SMS/SQLS-S scores presented with p-values; correlation coefficients reported (Results, lines 101–145). |
| **Discussion** | | |
| Key results | 18 | Summarized in Discussion (lines 146–245) and Conclusion (lines 246–260). |
| Limitations | 19 | Cross-sectional design and generalizability discussed in Abstract and Discussion (lines 25–30, |

| | | |
|---|---|---|
| | | 215–220). |
| Interpretation | 20 | Detailed in Discussion section (lines 146–245). |
| Generalisability | 21 | Discussed in Discussion (lines 215–220). |
| **Other information** | | |
| Funding | 22 | Not specified in manuscript excerpt; include if applicable. |

*Give information separately for cases and controls in case-control studies and, if applicable, for exposed and unexposed groups in cohort and cross-sectional studies.

**Note:** An Explanation and Elaboration article discusses each checklist item and gives methodological background and published examples of transparent reporting. The STROBE checklist is best used in conjunction with this article (freely available on the Web sites of PLoS Medicine at http://www.plosmedicine.org/, Annals of Internal Medicine at http://www.annals.org/, and Epidemiology at http://www.epidem.com/). Information on the STROBE Initiative is available at www.strobe-statement.org.
