# Supplementary material for: Correlatıonal effect of sexual myths on sexual qualıty of lıfe in pregnancy: a cross-sectıonal study
Source: Sex Med. 2026 Apr 20;14(3):qfag023. doi: 10.1093/sexmed/qfag023 (PMC13092729; doi:10.1093/sexmed/qfag023)
Supplement: Supplementary-Material_qfag023 [file supplementary-material_qfag023.zip › Tugce_Sevde_ve_Hilal_etik_izni_qfag023.pdf]

T.C.
TOKAT GAZİOSMANPAŞA ÜNİVERSİTESİ
SOSYAL VE BEŞERİ BİLİMLER ARAŞTIRMALARI
ETİK KURULU KARARLARI

KARAR TARİHİ	OTURUM NO	KARAR SAYISI
26.11.2024	19	01-52

Üniversitemiz Sosyal ve Beşerî Bilimler Araştırmaları Etik Kurulu Başkanı Prof. Dr. Nail YILDIRIM Başkanlığında toplandı.

KARAR 19.03- Sağlık Bilimleri Fakültesi Dekanlığının 13.11.2024 tarih ve 497338 sayılı yazısı görüşüldü.

Aşağıda bilgileri yer alan araştırmacıların yapmak istediği uygulamaların ve kullanacağı veri toplama araçlarının etik açıdan uygunluğuna oy birliği ile karar verildi.

ÇALIŞMANIN TÜRÜ	Öğretim Üyesi Araştırması
BAŞLIK	Gebelikte Deprem Anksiyetesinin Doğum Korkusuna Etkisi
TEZ YÜRÜTÜCÜSÜ/ ÇALIŞMANIN YAZARI	Dr. Öğr. Üyesi Gizem ÇITAK<br>Hilal BULDUK (Ebelik Anabilim Dalı)
RAPORTÖR GÖRÜŞÜ	OLUMLU

KARAR 19.04- Sağlık Bilimleri Fakültesi Dekanlığının 13.11.2024 tarih ve 497338 sayılı yazısı görüşüldü.

Aşağıda bilgileri yer alan araştırmacıların yapmak istediği uygulamaların ve kullanacağı veri toplama araçlarının etik açıdan uygunluğuna oy birliği ile karar verildi.

ÇALIŞMANIN TÜRÜ	Öğretim Üyesi Araştırması
BAŞLIK	Gebelikte Cinsel Mitlerin Cinsel Yaşam Kalitesine Etkisi
TEZ YÜRÜTÜCÜSÜ/ ÇALIŞMANIN YAZARI	Dr. Öğr. Üyesi Gizem ÇITAK<br>Tuğçe GÖRÜCÜ (Ebelik Anabilim Dalı)
RAPORTÖR GÖRÜŞÜ	OLUMLU

KARAR 19.05- Sağlık Bilimleri Fakültesi Dekanlığının 15.11.2024 tarih ve 497666 sayılı yazısı görüşüldü.

Aşağıda bilgileri yer alan araştırmacıların yapmak istediği uygulamaların ve kullanacağı veri toplama araçlarının etik açıdan uygunluğuna oy birliği ile karar verildi.

ÇALIŞMANIN TÜRÜ	Öğretim Üyesi Araştırması
BAŞLIK	Doğum Sonu Dönemde Emzirme Mitlerinin Emzirme Öz Yeterliliğine Etkisi
TEZ YÜRÜTÜCÜSÜ/ ÇALIŞMANIN YAZARI	Dr. Öğr. Üyesi Gizem ÇITAK<br>Sevde ÇETİNKAYA (Ebelik Anabilim Dalı)
RAPORTÖR GÖRÜŞÜ	OLUMLU

KARAR 19.52- Gündemde görüşülecek başka madde olmadığından oturuma son verild

KARAR TARİHİ	OTURUM NO	KARAR SAYISI
26.11.2024	19	01-52

Prof. Dr. Nail YILDIRIM
Etik Kurul Başkanı
(İmza)

Prof. Dr. Mehmet Serkan UMUZDAŞ
Başkan Yardımcısı
(İmza)

Prof. Dr. Emine ÖĞÜK
Üye
(İmza)

Prof. Dr. Mehmet KARGÜN
Üye
(İmza)

Prof. Dr. Muhittin DEMİRAY
Üye
(İmza)

Doç. Dr. Tuğba KILIÇER
Üye
(İmza)

Doç. Dr. Hüseyin Baha ÖZTUNÇ
Üye
(İmza)
